# Semantic Queries Expedite MedDRA Terms Selection Thanks to a Dedicated User Interface: A Pilot Study on Five Medical Conditions

**DOI:** 10.3389/fphar.2019.00050

**Published:** 2019-02-06

**Authors:** Julien Souvignet, Gunnar Declerck, Béatrice Trombert-Paviot, Hadyl Asfari, Marie-Christine Jaulent, Cédric Bousquet

**Affiliations:** ^1^Laboratoire d’Informatique Médicale et d’Ingénierie des Connaissances en e-Santé, INSERM, Sorbonne Université, Université Paris 13, Paris, France; ^2^EA 2223 Costech (Connaissance, Organisation et Systèmes Techniques), Centre de Recherche, Sorbonne Universités, Université de Technologie de Compiègne, Compiègne, France; ^3^Public Health and Medical Information Unit, University Hospital of Saint-Etienne, Saint-Étienne, France

**Keywords:** adverse drug reaction, MedDRA, SNOMED CT, ontology, pharmacovigilance

## Abstract

**Background:** Searching into the MedDRA terminology is usually limited to a hierarchical search, and/or a string search. Our objective was to compare user performances when using a new kind of user interface enabling semantic queries versus classical methods, and evaluating term selection improvement in MedDRA.

**Methods:** We implemented a forms-based web interface: OntoADR Query Tools (OQT). It relies on OntoADR, a formal resource describing MedDRA terms using SNOMED CT concepts and corresponding semantic relations, enabling terminological reasoning. We then compared time spent on five examples of medical conditions using OQT or the MedDRA web-based browser (MWB), and precision and recall of the term selection.

**Results:** OntoADR Query Tools allows the user to search in MedDRA: One may enter search criteria by selecting one semantic property from a dropdown list and one or more SNOMED CT concepts related to the range of the chosen property. The user is assisted in building his query: he can add criteria and combine them. Then, the interface displays the set of MedDRA terms matching the query. Meanwhile, on average, the time spent on OQT (about 4 min 30 s) is significantly lower (−35%; *p* < 0.001) than time spent on MWB (about 7 min). The results of the System Usability Scale (SUS) gave a score of 62.19 for OQT (rated as good). We also demonstrated increased precision (+27%; *p* = 0.01) and recall (+34%; *p* = 0.02). Computed “performance” (correct terms found per minute) is more than three times better with OQT than with MWB.

**Discussion:** This pilot study establishes the feasibility of our approach based on our initial assumption: performing MedDRA queries on the five selected medical conditions, using terminological reasoning, expedites term selection, and improves search capabilities for pharmacovigilance end users. Evaluation with a larger number of users and medical conditions are required in order to establish if OQT is appropriate for the needs of different user profiles, and to check if conclusions can be extended to other kinds of medical conditions. The application is currently limited by the non-exhaustive coverage of MedDRA by OntoADR, but nevertheless shows good performance which encourages continuing in the same direction.

## Introduction

Terminologies have been used for several centuries (Graunt, 1977) in order to describe or code qualitative biomedical data, allowing classification, and statistics. Classical terminologies are still in use as standards for coding, indexing, etc. However, some authors have suggested that classical terminologies are not sufficient for representing the meaning of terms in order to enable advanced treatment of clinical data in modern health structures. These authors have argued for replacing classical terminologies by formal terminological systems (Cimino, 1998). One solution is to use description logics (DL) and standards such as OWL (Web Ontology Language) to describe the meaning of terms and benefit from the properties of formal languages by enabling semantic processing of data.

Such formalization aims “to resolve vagueness and ambiguity, and to detect redundancy, to assist in the assignment of concepts to appropriate multiple classes” (Cimino et al., 1994), and to meet requirements of modern information systems through the explicit representation of the meaning of terms. Formal representation of a system brings harmonization (Rodrigues et al., 2009), semantic interoperability (Liyanage et al., 2015), facilitates computational processing and reasoning algorithms (Cimino et al., 1994), and intends to improve data quality (Liaw et al., 2013). But, in doing so, systems are increasingly complex and large, adding undesirable intricacy on the user side when interacting with the terminology [when entering, synthetizing, analyzing or searching data or implementing models or methods (Cimino, 2006)]. Simplification of operating procedures with specific tools and interfaces that meet users’ needs is a way to reconcile users with such systems (Rector et al., 1998).

Since 12 years, two authors (CB and M-CJ) are conducting a pharmacovigilance research program that aims to demonstrate the benefits of adding formal definitions to terminologies used to code adverse drug reaction (ADR) (Bousquet et al., 2005; Henegar et al., 2006). Indeed, reconciliation between MedDRA (Medical Dictionary for Regulatory Activities) and SNOMED CT (SNOMED – Clinical Terms) is desirable for standard harmonization (Richesson et al., 2008). For this purpose, we have developed OntoADR, a semantic resource of ADRs (Souvignet et al., 2016b). OntoADR expresses the meaning of the terms of MedDRA terminology, using formal definitions and is mainly used for pharmacovigilance purposes (Souvignet et al., 2016a).

**FIGURE 1:**
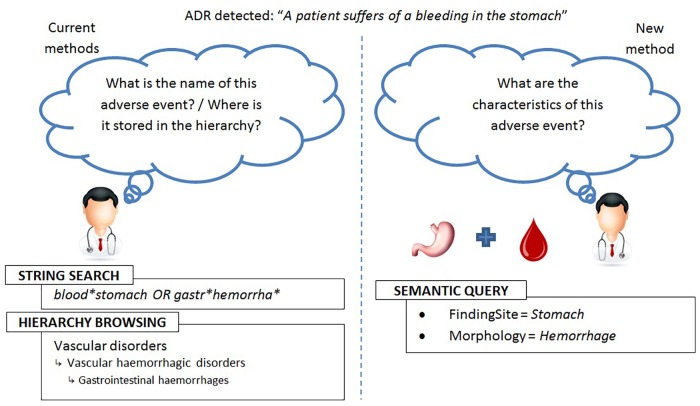
Comparison between string search and hierarchical browsing (left) with semantic queries (right).

Searching into the MedDRA terminology is limited to two search methods: a hierarchical search, or a string search (see Figure 1, left), as recommended in the MedDRA introductory guide and implemented in softwares such as MedDRA Browser (MSSO, 2018). In previous work, we presented results of querying MedDRA using a third method: semantic querying through the OntoADR resource (Declerck et al., 2012). Using the Protégé ontology editing software (Musen and Protégé Team, 2015), we aimed to search terms, and were able to build queries and perform semantic reasoning with encouraging results. However, such an approach to query MedDRA (through OntoADR) turned out to be tedious. As we proved our ability to improve search in MedDRA using knowledge engineering techniques, we decided to take a more pragmatic approach: analyzing the practices of users and their expectations when querying MedDRA.

When using a terminology such as MedDRA for coding or searching for cases in ADR databases, the most important identified users’ needs are: (1) improving term searches by making it easier and faster, and (2) searching for similar terms corresponding to the same medical condition using defining criteria. Adding formal definitions to MedDRA terms would address these problems by adding a new search method: semantic reasoning (see Figure 1, right; Bousquet et al., 2014). The domain lacks a dedicated interface for potential users who have no training in computer science and would be uncomfortable using a tool such as Protégé. Interface simplification and user-friendliness of a dedicated query form, in response to end-users needs, will make this method for searching in MedDRA accessible to new users, complement existing methods, demonstrate the relative advantage of a formal system, and especially, facilitate MedDRA search.

The ability for non-experts to query ontologies (or any knowledge resource) without having to learn a particular query language is a step toward scientific popularization of formal systems. To date, four approaches for providing access to concepts in an ontology are available (see Table 1).

**Table 1:** The four different approaches for providing access to concepts in an ontology.

Approach	Example(s)	Comment
Formal query languages	SPARQL (SPARQL Protocol and RDF Query Language) The W3C SPARQL Working Group (2018) or Manchester OWL Syntax (Horridge et al., 2006)	These languages require a substantial training of users and minimum knowledge about ontology structures.
Natural language interfaces (NLI)	Ginseng (Kaufmann and Bernstein, 2010) or Sparklis (Ferré, 2017)	They transform natural language queries into formal languages. These interfaces are user-friendly but despite their belonging to natural language processing, queries in NLI remain constrained to a given syntax.
Graphical representation interfaces	PharmARTS (Alecu et al., 2007)	Users can interactively manipulate a graphical representation of ontology elements to build a query. Such interface relies on user search capacities and is usually completed by a traditional form.
Forms-based query interfaces	Rhizomer (Brunetti et al., 2013) or PepeSearch (Vega-Gorgojo et al., 2016)	They describe queries using classical form inputs such as dropdown lists and checkboxes. Forms-based interfaces have the advantage to hide the complexity of formal languages while maintaining fair reasoning capabilities and good readability for user.

We assume that forms-based interfaces can result in an even more user-friendly interface in pharmacovigilance. This paper investigates the hypothesis that such interface would lower the complexity of querying and corresponds to the user’s expectations for searching MedDRA terms. We implemented semantic queries in a prototype named OntoADR Query Tools (OQT), a dedicated forms-based interface to query MedDRA terminology through semantic criteria.

Our objective was to demonstrate that such interface improves search in MedDRA compared to classical approaches.

## Materials and Methods

### Data Sources

#### MedDRA

MedDRA is a standard controlled terminology created to describe ADRs (Brown et al., 1999). MedDRA terms are hierarchically organized in five levels. A commonly used level is the preferred terms (PT) level. MedDRA also includes manual groupings of MedDRA PTs related to a medical condition or area of interest: Standardized MedDRA Queries (SMQ) (Mozzicato, 2007). We used MedDRA version 17.0 (March 2014) which included 20,559 PTs.

#### SNOMED CT

SNOMED – Clinical Terms is a large-scale medical resource terminology with a great depth and coverage of healthcare data. SNOMED CT is a formal system representing its semantics with DL EL++ (Baader et al., 2005). Thanks to our UMLS (Unified Medical Language System) license we accessed to SNOMED CT Release Files, and we used a SNOMED CT 20130131 version (January 2013) in this study.

#### OntoADR

OntoADR is a semantic resource we created to describe the meaning of MedDRA terms in a formal language. The semantic properties used to define ADRs have been selected from SNOMED CT. Today about 13,000 MedDRA terms (63%) have been defined in OntoADR using automated methods (Bousquet et al., 2014). Moreover, 1,935 terms related to 23 safety topics defined by Trifirò et al. (2009) were manually curated by an expert in order to be completely defined in OntoADR (Souvignet et al., 2014; Asfari et al., 2016).

### Terminological Reasoning

A semantic approach makes easier the translation of the pharmacovigilance’ thinking into a computable representation: pharmacovigilance specialists have the implicit knowledge that “gastric hemorrhage” is related to a bleeding in the stomach, while the semantic resource has an explicit knowledge specifying that:

“gastric hemorrhage” has Finding Site “Stomach”
“gastric hemorrhage” has Associated Morphology “Hemorrhage.”

This modeling of semantic relations between MedDRA ADR terms and medical concepts (such as body structure concepts) from SNOMED CT is explicit in the OntoADR resource.

### Interface Requirements

Software requirements are intended to fit how an end-user usually performs the following tasks: (1) Search for MedDRA term using either classical methods (id, string match, hierarchical browsing) or the new method (semantic criteria); (2) (Optional) Use additional features to adapt the resulting matching MedDRA terms list, exclude terms, etc.; (3) Select and export results (e.g., for signal detection tools).

We agreed on the following simplified technical requirements: (1) Simultaneous access to the interface allowing multiple users to query the resource; (2) Easy update of the underlying formal representation of ADRs; (3) Deployment on any operating system (Multi-platform); (4) Low latency responses; (5) other requirements mainly about ergonomic issues (see below).

### Evaluation Protocol

To evaluate the performance of OQT and assess its added value, we compared it with existing software. Today, to our knowledge, there is no similar software, that is to say, capable of searching MedDRA terms by their semantic characteristics. Nevertheless, there are (1) tools to search MedDRA terms using string search or hierarchical browsing; and (2) tools capable of managing and inferring semantic knowledge.

To compare OQT with an existing software to search MedDRA terms, we chose the MedDRA browser (MedDRA browser) because: (a) MedDRA browser is dedicated to the search of MedDRA terms and (b) MedDRA browser is the “official” tool for searching the terminology made available by MedDRA developers (International Conference on Harmonization). As OQT is a web application, we chose the online version: MedDRA web-based browser (MWB). The “web” version of MedDRA browser has the same features as the “desktop” version.

To compare OQT with an existing software able to manage semantic knowledge, we chose Protégé (Stanford Center for Biomedical Informatics Research, 2018) because: (1) Protégé is the reference software in terms of semantic knowledge base management; (2) Protégé allows the creation of semantic queries similar to OQT.

While MWB is a classical search software, accessible by novices and not requiring any technical skills, Protégé requires expertise about query languages and semantic knowledge. We judged that executing a query in Protégé would have necessitated extensive training and was unachievable for users who had no experience of semantic querying. Moreover, the comparison of results with Protégé and OQT should be limited (or exactly the same), as for a given query, they are using similar semantic inference engine. We decided to limit the Protégé comparison to some factual observations (see section “Results”).

#### User’s Profiles

In order to address potential user profile range, we selected eight healthcare professionals (see Table 2). We decided to select users according to the usual profiles in regional pharmacovigilance centers in France. This is why our sample consists of physicians and pharmacists on one hand, and juniors and seniors on the other hand. Additionally we included two physicians who had no previous knowledge of coding with MedDRA, but had experience of coding patient stays with ICD-10 in the case mix database of a university hospital.

**Table 2:** Profession, experience, and field of experience of the eight users.

User	Profession	Experience	Field of experience
#1	Pharmacist	Junior	Pharmacovigilance
#2	Physician	Senior	Pharmacovigilance
#3	Physician	Senior	Public health (no experience of MedDRA)
#4	Pharmacist	Junior	Pharmacovigilance
#5	Physician	Senior	Pharmacovigilance
#6	Physician	Senior	Pharmacovigilance
#7	Pharmacist	Senior	Pharmacovigilance
#8	Physician	Junior	Public health (no experience of MedDRA)

#### Test Content

We chose five safety topics; none of them had an exact corresponding reference grouping in MedDRA: (#1) “Myocardial Infarct,” (#2) “Acute Pancreatitis,” (#3) “Venous Thrombo-embolism,” (#4) “Peripheral Demyelination,” and (#5) “Upper Gastrointestinal Bleeding,” Among these topics, (#4) was expected to consist of very few terms (5 PTs), while the three others were larger (15–30 PTs) and (#3) was especially large (50 PTs). We prepared instructions for each safety topic with a definition and lists of inclusion/exclusion criteria (Supplementary Appendix A).

A gold standard was designed by an expert in pharmacovigilance for each topic. He selected the PTs in MedDRA without knowledge of OQT and MWB results. Supplementary Appendix B provides lists of MedDRA terms for these five safety topics.

#### Test Procedure

OntoADR Query Tools and MWB were presented to the users during 10 min, and demonstrated with a fictive example. There was no training period. Users also had a paper printed tutorial that we designed for each software, summarizing the main features with screen captions.

The five safety topics were presented to users in the same order, from (#1) to (#5). In this cross-over study, each user tested both softwares. For each topic, evaluation was divided in two consecutive periods. Half of the users started with MWB and half with OQT in the first period. Softwares were switched for the second period, in order to avoid a training effect on a safety topic.

For MWB, users were instructed to use either the search form or the hierarchical browser and were allowed to select individual terms or existing groupings. We chose to avoid the use of SMQs, as the evaluation consisted in the comparison of two tools for creating a list and not for using groupings already built by experts. For OQT no special instructions were given.

The search was timed and the terms selected by users were recorded for further comparison and analysis. With this data we computed several evaluation criteria per topic: time spent and number of correct and incorrect terms using the statistical value of precision and recall.

$$\mathrm{Precision}=\frac{\mathrm{relevant terms}\;\cap \;\mathrm{retrieved terms}}{\text{retrieved terms}}\mathrm{and}$$

(1) $$\mathrm{Recall}=\frac{\mathrm{relevant terms}\;\cap \;\mathrm{retrieved terms}}{\mathrm{total relevant terms}}$$

In order to compare a similar lists of terms, we used only PTs. When hierarchically related terms were selected we listed their equivalent PT(s).

Additionally, we used the System Usability Scale (SUS) (Brooke, 1996), a tool for measuring the usability of a system through a 10-question survey with five response options (from Strongly agree to Strongly disagree). We asked users to fill the SUS survey after the whole evaluation for the two softwares. We also asked the users to give a score of “perceived usefulness” and a score (from 0 to 5) of “perceived ease of use” (Davis, 1989) for each tool/safety topic.

We used ANOVA to compare time spent, precision, recall, “perceived usefulness,” and “perceived ease of use” on a data file consisting of 80 observations (8 users × 2 tools × 5 safety topics) by adjusting on the user effect. Due to the low user number, we used a non-parametric test (Wilcoxon) to test the difference of scores between OQT and MWB relative to the different users.

## Results

### Software Description

#### Technical Architecture

We chose a centralized server and a Web interface allowing multiple users to query the resource simultaneously. We used an AMP-server (Apache+MySQL+PHP). MySQL supports about two millions of records necessary to represent MedDRA, SNOMED CT, and OntoADR.

#### Current Interface

**FIGURE 2:**
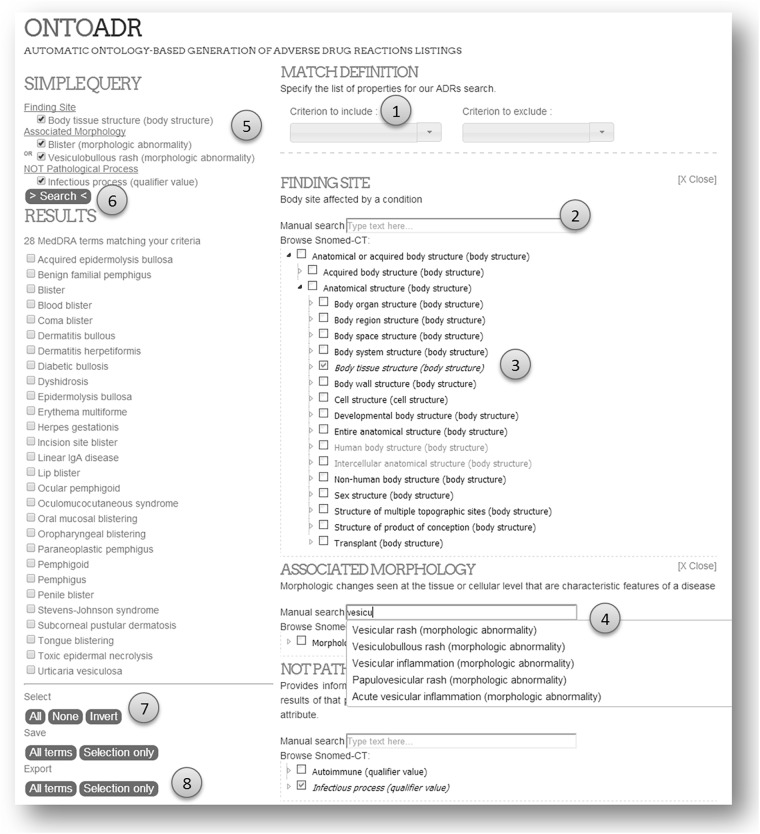
Screenshot of the OntoADR Query Tools interface.

The main interface of OQT is dedicated to search MedDRA terminology by performing semantic queries – see Figure 2. The screen initially presents a list of criteria available (1) for defining medical conditions (e.g., “Finding Site”). The form is adaptive, for a given selected criterion; a dedicated part appears contextually: an input text box (2) and a SNOMED CT browser limited to the allowed branch (3) (e.g., “Anatomical or acquired body structure”), when typing automatic completion activates and displays proposals of potential concepts (4). The query is synthesized on the top left of the screen (5). A submit button initiates a server-side computation, returning a list of MedDRA terms matching the criteria (6). The user may select terms (7) and export them (8) in one click.

#### Combination of Queries

Queries can be combined to be as complex as needed. From two lists of MedDRA terms already established, this tool can combine them using the set operators: OR, AND, XOR, and NOT to generate new sets of terms, respectively, Union, Intersect, Except, and Negation sets.

#### Automatic Completion and Suggestions

To accelerate interface suggestions when typing, we filtered the proposals made by OQT using ontological ranges. For example: If the user is looking for a finding site starting with “liv,” the software will make suggestions such as “liver,” and not “livedo” nor “lividity.”

#### SNOMED CT Browsing

To help the user finding a concept, we developed a SNOMED CT browser. The hierarchical tree is dynamically loaded on demand and is limited to the ontological range related to the selected relation. For example, hasFindingSite has for range “Anatomical or acquired body structure” subconcepts and only these allowable concepts are displayed.

#### Exporting Results

Users can export their results (created groups of PTs) in order to enable to import them in other tools, e.g., signal detection softwares. We set up a system of checkboxes to allow selection of the relevant terms. Buttons allow selection of all terms, deselection of them all, or invertion of the current selection.

Two storage methods are proposed: one using the Web browser capabilities to save lists of MedDRA terms directly in the storage areas of the browser in order to keep them until next sessions. The other method allows exporting the created lists in a file using CSV (comma separated values) format.

#### Ergonomics

We focused on reducing interaction by (1) replacing the handwriting of the query with a combo system and automatic completion; (2) reducing the number of mouse moves and clicks (using autofocus, e.g., automatic positioning of the cursor in the expected input box following a user event such as selecting a criterion); (3) reducing load times (using Asynchronous JavaScript and XML) without reloading pages; (4) exporting results in one click; and (5) allowing edition of the query by toggling criteria.

#### Speed Optimization

To speed up selection process among SNOMED CT, we have listed only concepts that are used in OntoADR and, so, may be retrieved by semantic queries. For example, in OntoADR with the current state of advancement in formal definitions, no MedDRA term is defined with the SNOMED CT finding site “Cerebellar white matter structure,” nor any of its subsumed elements such as “Structure of fastigiobulbar projection” or “Structure of inferior medullary velum.” When searching or browsing SNOMED CT, all these elements are visible but not selectable by users. When querying, the system does not spend time dealing with these elements, significantly speeding up the calculation process.

### Software Evaluation

#### Comparison With MedDRA Browser

**FIGURE 3:**
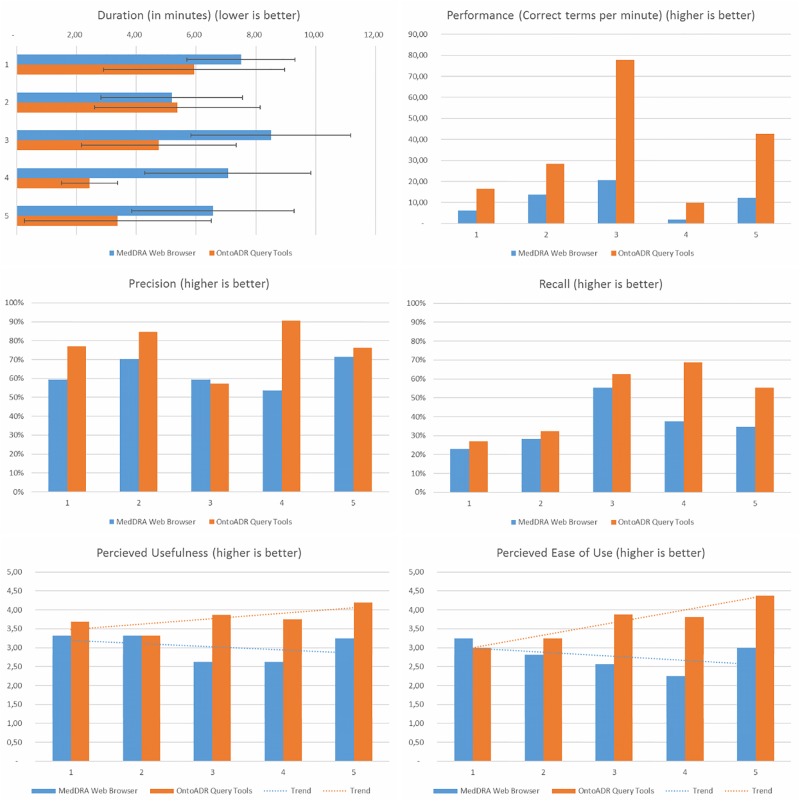
Barcharts showing measures of duration, performance, precision, recall, perceived usefulness, and perceived ease of use for the five safety topics.

Figure 3 presents the main results of the comparison of OQT and MWB. The use of OQT allows improving recall (+37%, *p* = 0.01) and precision (+24%, *p* = 0.02). Meanwhile, on average, the time spent on OQT (about 4 min 30 s) is significantly lower (−35%, *p* < 0.001) than time spent on MWB (about 7 min). Computed “performance” (correct terms found per minute) is more than three times better with OQT than with MWB.

On average with MWB, users make 1.95 string searches and use the hierarchy 82.5% of the time before having a satisfying result. For OQT, only one query is necessary 80% of the time. We observed that users had no major difficulties identifying the relevant SNOMED CT terms in OQT.

The “perceived usefulness” and “perceived ease of use” is better in OQT than in MWB (*p* < 0.001), and the difference is increasing over time. The results of the SUS were 62.19 for OQT and 41.25 for MWB (*p* = 0.06). According to Bangor et al. (2009) the closest adjective in terms of user-friendliness for OQR is “Good,” while MWB is “Poor.” Table 3 depicts the difference between the SUS scores associated with the users’ answers between OQT and MWB, where a positive difference indicates a favorable appreciation of OQT compared to MWB. The mean difference was superior to zero for each question suggesting a preference for OQT, but the difference was not significant for each question. The total of differences was superior for 6 users among 8 which also favors OQT. Among the 10 questions of the SUS, three were associated to a score significantly different between OQT and MWB: (1) “I think that I would like to use this system frequently” (applies to OQT, difference = +2.9, *p* = 0.048); (2) “I found the system unnecessarily complex” (applies to MWB, difference = +5.0, *p* = 0.049); and (3) “I found the system very cumbersome to use” (applies to MWB, difference = +5.4, *p* = 0.039).

**Table 3:** Difference between the SUS scores associated to the users’ answers between OQT and MWB (a positive difference indicates a favorable appreciation of OQT compared to MWB).

Question	User #1	User #2	User #3	User #4	User #5	User #6	User #7	User #8	Mean	*p*
I think that I would like to use this system frequently	0	0	2.5	2.5	7.5	2.5	2.5	0	2.9	0.048^∗^
I found the system unnecessarily complex	2.5	0	5	2.5	7.5	7.5	7.5	−2.5	5.0	0.049^∗^
I thought the system was easy to use	2.5	−2.5	5	5	5	10	2.5	−2.5	4.2	0.075
I think that I would need the support of a technical person to be able to use this system	0	0	0	2.5	2.5	2.5	0	−2.5	1.3	0.424
I found the various functions in this system were well integrated	0	2.5	2.5	0	0	2.5	2.5	0	1.7	0.072
I thought there was too much inconsistency in this system	−2.5	2.5	2.5	2.5	0	5	2.5	−2.5	2.5	0.240
I would imagine that most people would learn to use this system very quickly	0	0	7.5	0	5	10	0	−2.5	3.8	0.201
I found the system very cumbersome to use	5	−2.5	7.5	7.5	5	7.5	7.5	−2.5	5.4	0.039^∗^
I felt very confident using the system	0	0	7.5	0	5	10	0	−2.5	3.8	0.201
I needed to learn a lot of things before I could get going with this system	0	−5	0	0	2.5	10	−2.5	0	0.8	1.000
Total	7.5	−5	40	22.5	40	67.5	22.5	−17.5	31.3	0.058

*^∗^p < 0.05.*

#### Comparison With Protégé

In Protégé, executing the query illustrated in Figure 2 would face multiple issues: (a) write the exact label corresponding to ontological entities (no typo or spelling mistake is tolerated, and the character case has to be exact), (b) respect syntactic rules of Manchester OWL syntax for DL queries, (c) have memorized the OntoADR relationships or be familiar with them, (d) use a non-intuitive and complex access path (select a reasoner, launch reasoning, wait for inference results, select DL query tab, write the query, click execute), and (e) manually write down labels of expected terms – as no export or copy/paste feature is available, and no id or other info is displayed.

In comparison typing “hasFindingSite some ‘Kidney Structure”’ in Protégé is 38-character long. The same query in OQT could be done with a 5-character input: “f”+<Enter>x+“ki”+<Enter>. Indeed, “f” will underline hasFindingSite as property, <Enter> validates this choice, the cursor is then automatically placed in the right input box, then typing “ki” will cause an automatic suggestion for “Kidney Structure” as a top proposal.

On calculation speed, generating a list of terms related to a given query in OQT is as fast as inferring the same list with the HermiT reasoner (Shearer et al., 2008) in Protégé (less than 1 s). Protégé constrains the user to additional time consuming steps that are not required in OQT: the time needed to read the file in Protégé (about 5 s), and the time to re-classify the whole ontology (about 30 s). Moreover, if there is any change in the semantic resource, a new inference is mandatory in Protégé, while minor revisions in OQT resource do not introduce additional time.

## Discussion

We showed that it is possible to develop an interface to query a semantic resource and select terms in MedDRA in an automated way. This kind of selection is performed manually in SMQs by MedDRA experts. Building custom groupings is difficult, which explains why the number of SMQs is limited, and they do not cover all medical conditions or may not have the desired specificity. Our interface could facilitate such selection of terms. The evaluation of OQT demonstrates that the interface is user friendly and users are able to select more terms with OQT than MWB, while at the same time these terms are often more accurate. As we performed this pilot study with five medical conditions, evaluation with a larger number of medical conditions is required in order to check if conclusions can be extended to other kinds of medical conditions. Indeed, the medical conditions were chosen by the authors whereas it would be preferable that they be chosen randomly, e.g., from a set of clinical situations that have been reviewed by a pharmacovigilance center in a given period of time, so as to have a wider range of use cases.

### Software Evaluation

We observed a trend of perceived Usefulness and perceived Ease of Use increasing with OQT, when evaluators select MedDRA terms for new safety topics, while it is slightly decreasing with the MWB. Consequently, we assume this phenomenon is related to a training effect with OQT. Additionally, durations were shorter with OQT after two topics, while the duration with MedDRA browser was stable over the time.

The users had no experience of OntoADR nor OQT, but the 6 pharmacovigilance specialists are knowledgeable about MedDRA organization, and previously used the MedDRA browser that is associated with the French pharmacovigilance databases. This constitutes a bias favoring MWB over OQT that we were not able to avoid.

The peripheral demyelination (#4) requires more specialized knowledge than other topics. In this case, OQT was especially efficient compared to MWB. This is probably due to the fact that evaluators were required to distinguish between medical conditions associated, respectively, to the peripheral nervous system and the central nervous system which necessitates special skills in neurology, while the association between medical conditions and finding sites is already encoded in OntoADR.

The average number of terms selected by each user was very heterogeneous. For example, with Venous thromboembolism, the number of terms ranged from 4 to 287 (78.38 ± 99.24) with MWB, and 15 to 79 (49.88 ± 23.59) with OQT. Indeed, some users chose to select only the precise condition, and some selected also investigation procedures or signs and symptoms for a given medical condition.

### Related Work

Developing innovative softwares, especially user interfaces, has been subject to multiple studies. Shneiderman has defined “8 Golden Rules of Interface Design” (Shneiderman and Ben, 2003): focusing on keeping terminological consistency in the interface, reducing the number of interactions, offering feedback, dialogs, simple error handling, easy reversal of actions, turning users as actors of the process, and reducing load of human short-term memory. Shackel and Dillon showed the importance of evaluating both technological acceptance and accessibility when releasing innovating software (Shackel, 1984; Dillon and Morris, 1996). Carroll dissected the psychology of users in human-computer interface interactions (Carroll, 1991) and showed the importance of the cognitive ergonomics. We followed these development principles which could explain why our tool is well received by users.

To our knowledge, no development has been realized on forms-based interfaces for knowledge engineering in pharmacovigilance. The only work, so far in the biomedical domain, is from Joubert et al. (1998) describing a software application where the user may select pairs of concepts and a relation between them, in order to build a conceptual graph and retrieve records in medical databases. However, it does not meet the needs of pharmacovigilance who aim to select medical terms. The SUS score for OQT suggests that forms-based interfaces show a better usability than existing systems.

Rogers (Rogers, 2010) described factors that can influence a decision to adopt or reject an innovation. In our case, the development of OQT helps to significantly improve the trialability of OntoADR (i.e., making reasoning OntoADR feasible by all).

### Usability

Several previous versions of OQT have been developed. Before running the test with eight users, OQT has previously been tested by three alpha testers. During the alpha test, a difficulty in selecting relevant OntoADR properties was observed: the label was difficult to understand by users. For example, some users indicated a finding site for laboratory procedures. Therefore, we chose to group properties by category (findings or procedures).

In the current version of OQT, one may object that search of MedDRA terms is replaced by an alternative search of SNOMED CT terms. Indeed the user has to specify SNOMED CT terms as a filter for the semantic queries. Search in SNOMED CT faces some difficulties inherent to its formalized labeling, that may not always appear intuitive. For example “Cerebellum” is not a SNOMED CT term and one has to specify Cerebellar_Structure in entry when he is searching for MedDRA terms such as “cerebellar ataxia.” We are currently working to improve user experience in such cases. Some additional difficulties remain with SNOMED CT, e.g., the results of “hasMorphology Hemorrhage” are not semantically equivalent to “hasDefinitionalManifestation Bleeding.” We could also draw some inspiration from the work of Bakhshi-Raiez et al. (2012) about a more advanced usability evaluation of our interface, especially for interaction with SNOMED CT concepts. Meantime, we plan to change some labels and better display the definition of each property in the user interface.

Despite these ergonomic issues related to SNOMED CT use, OQT did expedite searching of MedDRA terms. We still aim to improve ergonomics. Trialability and observability have been enhanced as the tool is accessible in any Web browser, and we created a short video tutorial for new users.

Our sample of users was too small to derive groups of users with similar behavior (depending on experience, or domain). We plan to further evaluate OQT with distinct groups of user profiles (15–20 users for each profile).

### Perspectives

OntoADR Query Tools interface is still evolving; multiple tools are currently in development such as a “common parenthood” feature that would list common properties from a set of terms, or a “query by example” feature that would suggest closest terms from a given selection.

OntoADR Query Tools is currently limited by the coverage of medical conditions by OntoADR and the number of semantic definitions validated by an expert. In this article we showed the applicability of the proposed method on five safety topics covering different pathologies and expect that OQT may be applicable to the whole MedDRA terminology when full coverage will be achieved.

In order for MedDRA users to benefit from the semantic queries we plan to present the current results to the MedDRA Maintenance and Services Organization (MSSO) that is in charge of delivering new versions of MedDRA, and to software providers that implement access to spontaneous reports databases.

#### Re-usability

The interface is currently only compatible with MedDRA and SNOMED CT. With minor modifications, it would be possible to query any terminology aligned with SNOMED CT, such as International classification of diseases (ICD) or SNOMED CT itself. In addition, SNOMED CT may be replaced by any terminology using the DL standard EL++ (such as NCI thesaurus, e.g.).

OQT communicates with the database using an internal API as web services. This programming interface is not documented yet, but it would be possible for external applications to launch queries using this web service. We also plan to explore possibilities offered by Common Terminology Services 2 standard (Object Management Group, 2018) for this API.

#### Legal Ramifications

OntoADR and OQT are not available to the public due to legal constraints in using MedDRA and SNOMED CT; indeed, both resources necessitate a valid license for using them.

## Conclusion

The tool we propose is a proof of concept that establishes the feasibility of our approach based on our initial assumption: performing MedDRA queries using terminological reasoning expedites term selection and improves search capabilities for pharmacovigilance end users. We claim that this such tool may be extended to other standard terminologies used in the medical domain such as ICD. OQT was developed following general design and ergonomics rules. So far, it has correct acceptance, accessibility, and feedbacks demonstrating that it has rather good ergonomics. Pharmacovigilance specialists are able to query MedDRA in a new way, without any training. We received a good reception and support interest for the approach with this tool, an interest we had not received in previous development phases. Adoption is on the right track and we plan to present the tool to pharmacovigilance specialists on a larger scale.

## Author Contributions

JS designed the OntoADR resource, implemented the OntoADR Query Tools, conducted the evaluation with users, and produced the analysis of the results. GD drafted the requirements for the design of the interface, was significantly involved in a first version of OntoADR, and was an alpha tester for OntoADR Query Tools. BT-P contributed to design the evaluation protocol and was an alpha tester. HA was an alpha tester and provided significant feedbacks. M-CJ was the supervisor of OntoADR initial version and provided several advices in all developmental steps. CB was the principal investigator of the conception of this new kind of interface for building custom groupings of ADR terms, contributed to drafting the evaluation protocol, and reviewed the results. JS and CB drafted the manuscript. All authors revised the article critically for important intellectual content and provided final approval of the version to be submitted.

## Conflict of Interest Statement

The authors declare that the research was conducted in the absence of any commercial or financial relationships that could be construed as a potential conflict of interest.
