# Supplementary material for: Semantic Queries Expedite MedDRA Terms Selection Thanks to a Dedicated User Interface: A Pilot Study on Five Medical Conditions
Source: Front Pharmacol. 2019 Feb 6;10:50. doi: 10.3389/fphar.2019.00050 (PMC6374626; doi:10.3389/fphar.2019.00050)
Supplement: Supplementary file 1 [file Table_1.DOCX]

# Appendix A. Definition and inclusion / exclusion criteria for each safety topic

# #1 Myocardial infarct

Definition: Myocardial infarction (MI) or acute myocardial infarction (AMI), commonly known as a heart attack, occurs when blood flow stops to part of the heart causing damage to the heart muscle. Most MIs occur due to coronary artery disease.

Inclusion: All kinds of infarction occurring in the myocardium; Commonly used blood tests include troponin and less often creatine kinase MB

Exclusion: Other kinds of ischaemic diseases without infarction

# #2 Acute pancreatitis

Definition: Acute pancreatitis is a sudden inflammation of the pancreas. It can have severe complications and high mortality despite treatment.

Inclusion: Acute inflammation of the pancreas, Related sign such as Cullen’s sign, Abnormal investigation result such as Lipase increased (enzyme frequently increased in acute pancreatitis)

Exclusion: Chronic pancreatitis

# #3 Venous thrombosis and embolism

Definition: A venous “thrombus” is a blood clot (thrombus) that forms within a vein. Thrombosis is a term for a blood clot occurring inside a blood vessel. When a blood clot breaks loose and travels in the blood, this is called a venous thromboembolism (VTE).

Inclusion: Thrombus and embolism that occurs in the “venous system”

Exclusion: Thrombus and embolism that occurs in the “arterial system”

# #4 Peripheral demyelination

Definition: The term demyelination describes a pathologic process of destruction of myelin-supporting cells, that is, oligodendrocytes and schwann cells in the central and peripheral nervous system, respectively and/or the myelin lamellae with relative preservation of axons. Depending on the primary site of demyelination in the nervous system it is divided into central demyelination involving the central nervous system and peripheral demyelination affecting the peripheral nervous system.

Inclusion: Demyelination in the peripheral nervous system

Exclusion: Demyelination in the central nervous system

# #5 Upper gastrointestinal bleeding

Definition: Occurrence of a haemorrhage originating from the upper gastrointestinal tract (from esophagus to Ligament of Treitz including esophagus, stomach and duodenum).

Inclusion: haemorrhage in the upper gastrointestinal tract, Symptoms and signs of UGIB (i.e. hematemesis, melena)

Exclusion: Bleeding in the lower gastrointestinal tract, Causes of UGIB that are not likely to be drug induced, e.g., variceal bleeding, or gastrointestinal malignancy
