# Supplementary material for: Semantic Queries Expedite MedDRA Terms Selection Thanks to a Dedicated User Interface: A Pilot Study on Five Medical Conditions
Source: Front Pharmacol. 2019 Feb 6;10:50. doi: 10.3389/fphar.2019.00050 (PMC6374626; doi:10.3389/fphar.2019.00050)
Supplement: Supplementary file 2 [file Table_2.DOCX]

## Appendix B. Content of reference groupings (gold standard for five safety topics)

| **#1 Myocardial infarct** |
| --- |
|  |
| Acute coronary syndrome |
| Acute myocardial infarction |
| Blood creatine increased |
| Blood creatine phosphokinase BB increased |
| Blood creatine phosphokinase increased |
| Blood creatine phosphokinase MB increased |
| Blood creatine phosphokinase MM increased |
| Coronary artery embolism |
| Coronary artery occlusion |
| Coronary artery reocclusion |
| Coronary artery thrombosis |
| Coronary bypass thrombosis |
| Electrocardiogram ST segment depression |
| Kounis syndrome |
| Myocardial infarction |
| Myocardial necrosis |
| Myocardial reperfusion injury |
| Myocardial stunning |
| Papillary muscle infarction |
| Post procedural myocardial infarction |
| Postinfarction angina |
| Silent myocardial infarction |
| Troponin I increased |
| Troponin increased |
| Troponin T increased |

| **#2 Acute pancreatitis** |
| --- |
|  |
| Alcoholic pancreatitis |
| Autoimmune pancreatitis |
| Cullen's sign |
| Cytomegalovirus pancreatitis |
| Hereditary pancreatitis |
| Ischaemic pancreatitis |
| Lipase abnormal |
| Lipase increased |
| Lupus pancreatitis |
| Oedematous pancreatitis |
| Pancreas infection |
| Pancreatic abscess |
| Pancreatic enlargement |
| Pancreatic enzyme abnormality |
| Pancreatic enzymes abnormal |
| Pancreatic enzymes increased |
| Pancreatic haemorrhage |
| Pancreatic necrosis |
| Pancreatic phlegmon |
| Pancreatic pseudocyst |
| Pancreatic pseudocyst drainage |
| Pancreatitis acute |
| Pancreatitis bacterial |
| Pancreatitis fungal |
| Pancreatitis haemorrhagic |
| Pancreatitis helminthic |
| Pancreatitis mumps |
| Pancreatitis necrotising |
| Pancreatitis relapsing |
| Pancreatitis viral |
| Pancreatorenal syndrome |

| **#3 Venous thrombosis and embolism** |
| --- |
|  |
| Axillary vein thrombosis |
| Cement embolism |
| Cerebral venous thrombosis |
| Deep vein thrombosis |
| Deep vein thrombosis postoperative |
| Embolism venous |
| Hepatic vein thrombosis |
| Jugular vein thrombosis |
| Mesenteric vein thrombosis |
| Ophthalmic vein thrombosis |
| Ovarian vein thrombosis |
| Paradoxical embolism |
| Pelvic venous thrombosis |
| Penile vein thrombosis |
| Portal vein thrombosis |
| Postpartum venous thrombosis |
| Protein S decreased |
| Pulmonary embolism |
| Pulmonary infarction |
| Pulmonary veno-occlusive disease |
| Pulmonary venous thrombosis |
| Renal vein embolism |
| Renal vein thrombosis |
| Retinal vein occlusion |
| Retinal vein thrombosis |
| Splenic vein thrombosis |
| Subclavian vein thrombosis |
| Thrombophlebitis |
| Thrombophlebitis migrans |
| Thrombophlebitis neonatal |
| Thrombophlebitis septic |
| Thrombophlebitis superficial |
| Thrombosed varicose vein |
| Vena cava embolism |
| Vena cava thrombosis |
| Venogram abnormal |
| Venous thrombosis |
| Venous thrombosis in pregnancy |
| Venous thrombosis limb |
| Venous thrombosis neonatal |

| **#4 Peripheral demyelination** |
| --- |
|  |
| Chronic inflammatory demyelinating polyradiculoneuropathy |
| Demyelinating polyneuropathy |
| Guillain-Barre syndrome |
| Peroneal muscular atrophy |

| **#5 Upper gastrointestinal bleeding** |
| --- |
|  |
| Dieulafoy's vascular malformation |
| Duodenal ulcer haemorrhage |
| Duodenitis haemorrhagic |
| Endoscopy upper gastrointestinal tract |
| Gastric antral vascular ectasia |
| Gastric haemorrhage |
| Gastric ulcer haemorrhage |
| Gastric ulcer haemorrhage, obstructive |
| Gastric varices haemorrhage |
| Gastritis alcoholic haemorrhagic |
| Gastritis haemorrhagic |
| Gastroduodenal haemorrhage |
| Gastroduodenitis haemorrhagic |
| Gastrointestinal angiodysplasia haemorrhagic |
| Gastrointestinal ulcer haemorrhage |
| Haematemesis |
| Haemorrhagic erosive gastritis |
| Mallory-Weiss syndrome |
| Melaena |
| Melaena neonatal |
| Neonatal gastrointestinal haemorrhage |
| Occult blood positive |
| Oesophageal haemorrhage |
| Oesophageal ulcer haemorrhage |
| Oesophageal varices haemorrhage |
| Oesophagitis haemorrhagic |
| Peptic ulcer haemorrhage |
| Ulcer haemorrhage |
| Upper gastrointestinal haemorrhage |
